# Complete chloroplast genome of novel *Adinandra megaphylla* Hu species: molecular structure, comparative and phylogenetic analysis

**DOI:** 10.1038/s41598-021-91071-z

**Published:** 2021-06-03

**Authors:** Huu Quan Nguyen, Thi Ngoc Lan Nguyen, Thi Nhung Doan, Thi Thu Nga Nguyen, Mai Huong Phạm, Tung Lam Le, Danh Thuong Sy, Hoang Ha Chu, Hoang Mau Chu

**Affiliations:** 1 Thainguyen University of Education, Thai Nguyen University, Thai Nguyen, 250000 Vietnam; 2 Institute of Biotechnology, Vietnam Academy of Science and Technology, Hanoi, 100000 Vietnam

**Keywords:** Biotechnology, Molecular biology, Plant sciences

## Abstract

*Adinandra megaphylla* Hu is a medicinal plant belonging to the *Adinandra* genus, which is well-known for its potential health benefits due to its bioactive compounds. This study aimed to assemble and annotate the chloroplast genome of *A. megaphylla* as well as compare it with previously published cp genomes within the *Adinandra* genus. The chloroplast genome was reconstructed using de novo and reference-based assembly of paired-end reads generated by long-read sequencing of total genomic DNA. The size of the chloroplast genome was 156,298 bp, comprised a large single-copy (LSC) region of 85,688 bp, a small single-copy (SSC) region of 18,424 bp, and a pair of inverted repeats (IRa and IRb) of 26,093 bp each; and a total of 51 SSRs and 48 repeat structures were detected. The chloroplast genome includes a total of 131 functional genes, containing 86 protein-coding genes, 37 transfer RNA genes, and 8 ribosomal RNA genes. The *A. megaphylla* chloroplast genome indicated that gene content and structure are highly conserved. The phylogenetic reconstruction using complete cp sequences, *matK* and *trnL* genes from Pentaphylacaceae species exhibited a genetic relationship. Among them, *matK* sequence is a better candidate for phylogenetic resolution. This study is the first report for the chloroplast genome of the *A. megaphylla*.

## Introduction

The chloroplast (cp) acts as a vital and essential organelle playing an indispensable role in several crucial biochemical processes and photosynthesis of plants^1^. The cp genome is uniparental inheritance and generally has a quadripartite structure including one large single-copy (LSC) region, one small single-copy (SSC) region, and two inverted repeat regions (IRs) of the same length^2^. In terms of gene structure and composition, the cp genome is more conserved, compared with nuclear and mitochondrial genomes^3^. These chloroplast DNA features were used by scientists to construct chloroplast DNA phylogenies, demonstrating to be greatly beneficial in the exploration of plant phylogenetic studies and more clarified taxonomic levels^4,5^. The whole chloroplast genome was reported as circular and its genes were a single evolutionary unit^6,7^. The chloroplast DNA genome sequencing in complex genome plants has been proven to be comparatively inexpensive and easy^8,9^.

*Adinandra* genus has been consumed as a traditional health tea beverage in China, and it has been described to have many curative effects, such as reduction of blood pressure, anti-inflammatory, antibacterial, antitumor, antitoxic, and analgesic effects^10,11^. Surprisingly, to date, limited reports have been published about *Adinandra* genus. *Adinandra megaphylla* Hu, belonging to the Adinandra genus, was first published in Bull. Fan Mem. Inst. Biol., Bot. 6: 172 (1935). The related information is about morphological descriptions^12^ and bioactivity assays^13^. A few species were used by molecular phylogenetics, including *A. dumosa*^14^; *A. elegans*, *A. formosan*, *A. lasiostyla*, *A.millettii*, *A. yaeyamensi*^12^; *A. millettii*, *A. angustifolia*^15^. To date, there have not been any studies on the genomes of *A. megaphylla* Hu, especially the chloroplast genome, this leads to the lack of information for estimating the phylogenetic relationships within *Adinandra* genus.

For the first time, we reported a new complete chloroplast genome of *A. megaphylla* Hu*,* and combined it with previously published Pentaphylacaceae complete chloroplast genomes data to visualize and evaluate the genome organization and phylogenetic relationships.

## Results

### Chloroplast genome assembly and annotation

**Figure 1:**
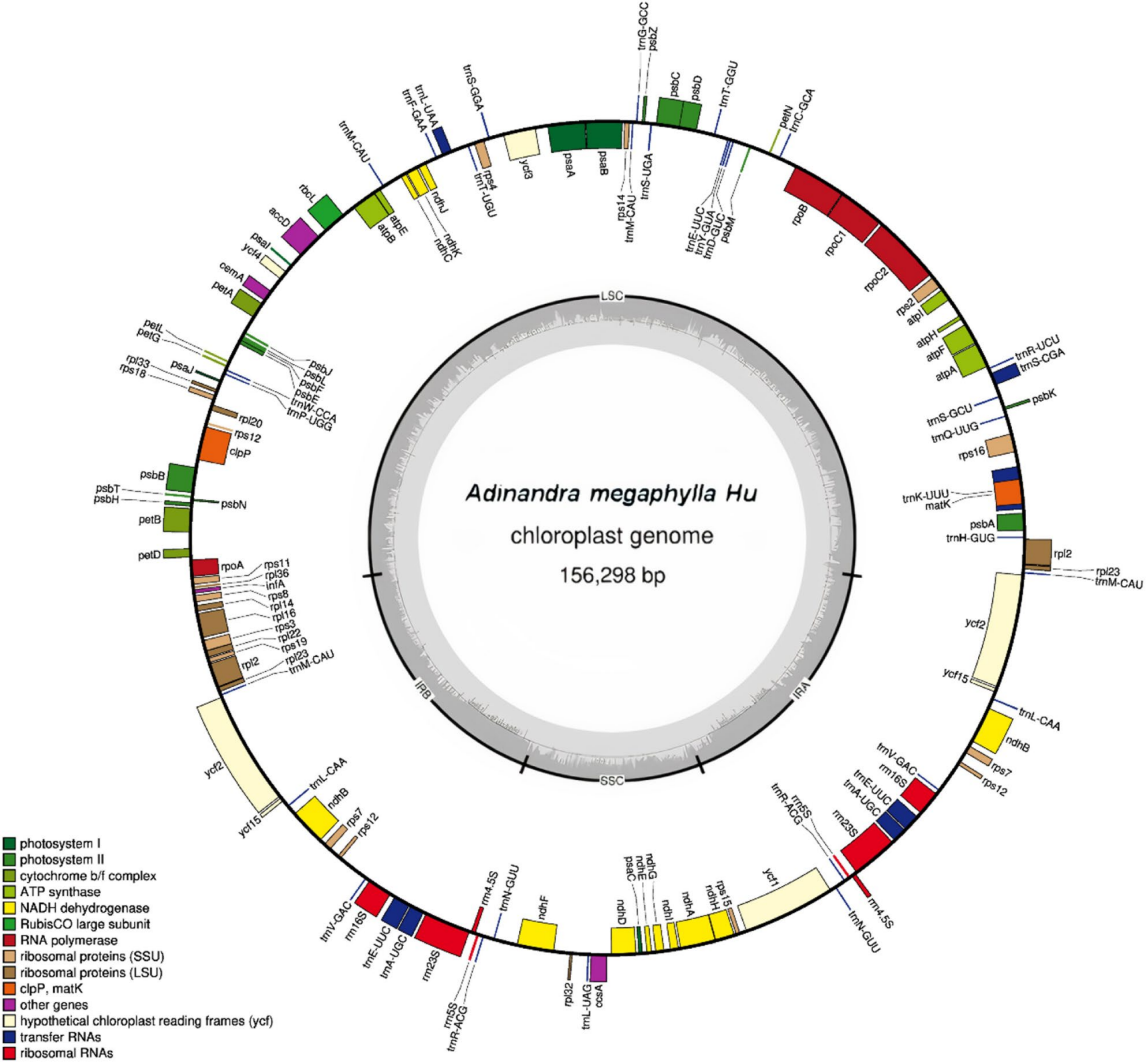
Chloroplast map of *A. megaphylla* Hu in Vietnam. Genes shown inside the circle are transcribed clockwise, whereas genes outside are transcribed counterclockwise. The light gray inner circle shows the AT content, the dark gray corresponds to the GC content.

Using the PacBio SEQUEL system, 29,815,452 bp of raw sequence data of the whole genome were generated from *A. megaphylla* Hu (Fig. 1). The mean read length is 2938 bp, the N50 contig size is 3594 bp and approximately 5% of the genomic genome belongs to the cp genome with 188 × coverage. The cp genome size of 156,298 bp of *A. megaphylla* Hu was derived from the assembly. As shown in most cp genomes, the assembled *A. megaphylla* Hu plastome exhibited the typical quadripartite structure comprising of the four regions, a pair of inverted repeats (IRs 26,093 bp), LSC (85,688 bp), and SSC (18,424 bp). Besides, the cp genome of *A. megaphylla* Hu contains 131 genes, and the percent of the GC content of the cp genome was 37.4% (Table 1).

**Table 1:** Summary of the chloroplast genome of *A. megaphylla* Hu species.

Genome features	*A. megaphylla* Hu
Genome size (bp)	156,298
LSC size (bp)	85,688
SSC size (bp)	18,424
IR size (bp)	26,093
GC content (%)	37.4
No. of genes	131
No. of PCGs	86
No. of tRNA	37
No. of rRNA	8

### Chloroplast genome annotation

The cp genome of *A. megaphylla* Hu includes 37 tRNA genes, 8 rRNA genes (16S, 23S, 5S, and 4.5S), and 86 protein-coding genes (Table 1). There were 123 genes assigned into three groups based on their functions. Regarding the photosynthesis-related gene category, there are 43 genes, containing genes encoding the large subunit of Rubisco related to the photosynthetic electron transport chain and putative NADPH dehydrogenase genes. Also, 68 genes were functioning in the transcription and translation processes. The majorities are tRNA genes, and the others are rRNA genes and genes encoding subunits of RNA polymerase and ribosome proteins. The remaining twelve genes with different functions are classified in the category of other genes, including five genes with known functions in fatty acid synthesis (*accD*), c-type cytochrome synthesis (*ccsA*), carbon metabolism (*cemA*), (*clpP*) proteolysis, and RNA processing (*matK*). Otherwise, five genes encoding for the conserved reading frames (*ycf1, ycf2, ycf3, ycf4, ycf15*) with unknown functions were annotated in the plastome. Eighteen genes (all 4 rRNA genes, 7 tRNA genes, 4 ribosomal protein-coding genes, 1 NADH-dehydrogenase protein-coding gene and 2 other genes) were annotated with two copies located in IR regions (Table 2). There are 18 cp genes harbored introns, among which 13 genes (*atpF, rpoC1, rpl2* (×2)*, ndhB* (×2)*, ndhA, petB, rpl16, trnA-UGC* (×2)*, trnC-ACA, trnE-UUC, trnK-UUU, trnL-UAA* and *trnS-CGA*) contained one intron, while 2 genes (*ycf3, clpP*) contained 2 introns (Table 2).

**Table 2:** Gene composition of *A. megaphylla* Hu chloroplast genome.

Category of genes	Group of genes	Name of genes
Photosynthesis	Subunits of ATP synthase	*atpA, atpB, atpE, atpF*^a^*, atpH, atpI*
	Subunits of NADH-dehydrogenase	*ndhA*^a^*, ndhB (*×*2)*^a^*, ndhC, ndhD, ndhE, ndhF, ndhG, ndhH, ndhI, ndhJ, ndhK*
	Subunits of cytochrome b/f complex	*petL, petB*^a^*, petG, petA, petD, petN*
	Subunits of photosystem I	*psaJ, psaC, psaA, psaI, psaB*
	Subunits of photosystem II	*psbA, psbB, psbC, psbD, psbE, psbF, psbH, psbJ, psbK, psbL, psbM, psbN, psbT, psbZ*
	Subunit of rubisco	*rbcL*
Transcription and translation	Large subunit of ribosome	*rpl14, rpl16*^a^*, rpl2 (*×*2)*^a^*, rpl20, rpl22, rpl23 (*×*2), rpl32, rpl33, rpl36*
	DNA dependent RNA polymerase	*rpoB, rpoA, rpoC1*^a^*, rpoC2*
	Small subunit of ribosomal proteins	*rps11, rps12, rps14, rps15, rps16*^a^*, rps18, rps19* (×2)*, rps2, rps3, rps4, rps7* (×2)*, rps8*
	rRNA genes	*rrn23S* (×2)*, rrn16S* (×2)*, rrn5S* (×2)*, rrn4.5S* (×2)
	tRNA genes	*trnA-UGC (*×*2)*^*a*^*, trnC-ACA*^*a*^*, trnC-GCA, trnD-GUC, trnE-UUC (*×*2)*^*a*^*, trnF-GAA, trnG-GCC, trnH-GUG, trnK-UUU*^*a*^*, trnL-CAA (*×*2), trnL-UAA*^*a*^*, trnL-UAG, trnM-CAU (*×*2), trnN-GUU (*×*2), trnP-UGG, trnQ-UUG, trnR-ACG (*×*2), trnR-UCU, trnS-CGA*^*a*^*, trnS-GCU, trnS-GGA, trnS-UGA, trnT-GGU, trnT-UGU, trnV-GAC (*×*2), trnW-CCA, trnY-GUA*
	Translational initiation factor	*infA*
Other genes	Subunit of acetyl-CoA-carboxylase (fatty acid synthesis)	*accD*
	c-type cytochrome synthesis gene	*ccsA*
	Envelope membrane protein (carbon metabolism)	*cemA*
	Protease	*clpP*^*b*^
	Maturase (RNA processing)	*matK*
	Conserved open reading frames	*ycf1, ycf2 (*×*2), ycf3*^*b*^*, ycf4, ycf15 (*×*2)*

Genes marked with the sign are the gene with a single (a) or double (b) introns and duplicated genes (×2).

### Repeat sequences and codon analysis

**Figure 2:**
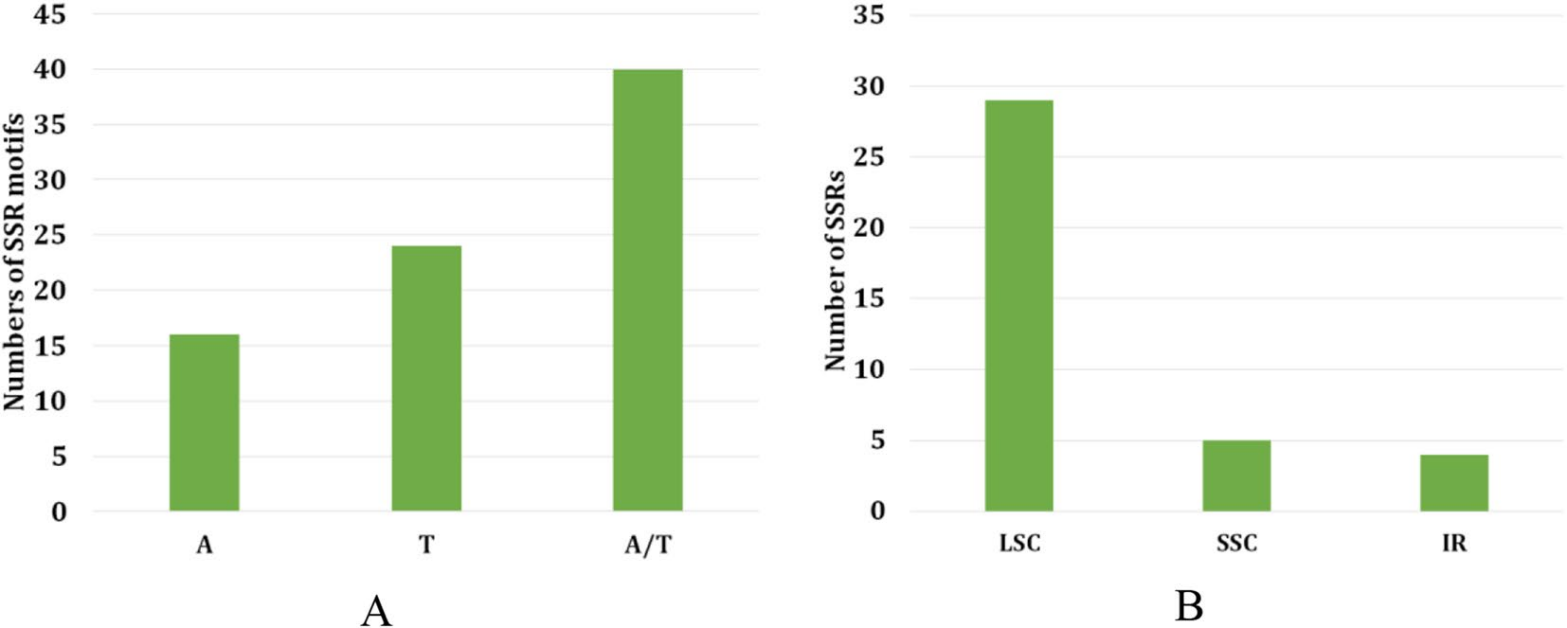
Analysis of single sequence repeats of plastome in *A. megaphylla* Hu. (**A**) Number of identified SSR sequence motifs; (**B**) Frequency of repeat types in LSC, SSC, and IR regions.

The total number of identified simple sequence repeats (SSRs) in the chloroplast genome of *A. megaphylla* Hu was 40. All repeats were mono repeats composed of A or T (size of 10–19) (Fig. 2A). There were no di-, tri-, tetra-, penta-, and hexa-nucleotide SSRs in the *A. megaphylla* Hu (Fig. 2B).

**Figure 3:**
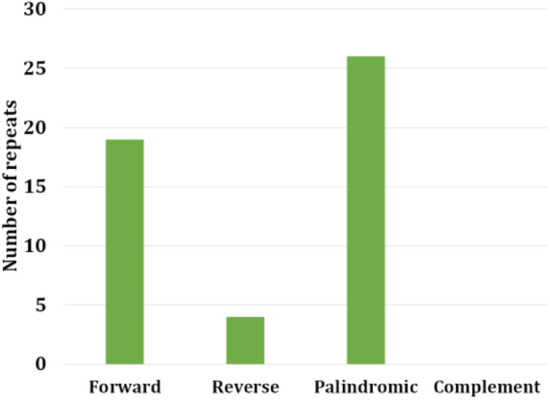
Repeat analysis on a genomic scale in *A. megaphylla* Hu.

The cp genome of *A. megaphylla* Hu was identified with 49 repeats consisting of 26 palindromic repeats, 19 forward and 4 reverse repeats. There were no complement repeats (Fig. 3). The smallest unit size of the repeat was 22 bp while the largest unit size was 62 bp. Most of the size of the repeats (72%) was higher than 30 bp.

The codon usage frequency of 64 protein-coding genes for three *Adinandra* species was evaluated. The total number of codons for protein-coding genes was 52,076 in those coding regions. G- and C-ending are found to be more frequent than their counterparts A and U (Table 3). Among the 20 amino acids, serine was the most abundant (number of codons encoding serine = 4975, 9.55%), leucine ranked second (number of codons encoding leucine = 4883, 9.37%), while the rarest one is tryptophan (677 codons, approximately 1.3%). Thirty codons were observed to be used more frequently than the expected usage at equilibrium (RSCU > 1) and thirty-one codons showed the codon usage bias: (RSCU < 1). Moreover, the frequency of use for the start codons AUG and UGG (methionine and tryptophan), as well as AUA (isoleucine) showed no bias (RSCU = 1).

**Table 3:** Relative synonymous codon usage (RSCU) for protein-coding genes in *A. megaphylla* Hu.

Codon	AA	ObsFreq	RCSU	Codon	AA	ObsFreq	RCSU	Codon	AA	ObsFreq	RCSU
UAG	*	672	0.73	AUC	I	1173	0.77	CGC	R	230	0.41
UGA	*	914	0.99	AUU	I	1853	1.22	CGG	R	361	0.65
UAA	*	1177	1.28	AAA	K	2071	1.33	CGU	R	401	0.72
GCA	A	382	1.06	AAG	K	1049	0.67	AGC	S	559	0.67
GCC	A	347	0.96	CUA	L	661	0.81	AGU	S	795	0.96
GCG	A	210	0.58	CUC	L	640	0.79	UCA	S	870	1.05
GCU	A	505	1.4	CUG	L	475	0.58	UCC	S	908	1.1
UGC	C	477	0.81	CUU	L	1019	1.25	UCG	S	638	0.77
UGU	C	697	1.19	UUA	L	934	1.15	UCU	S	1205	1.45
GAC	D	426	0.55	UUG	L	1154	1.42	ACA	T	680	1.12
GAU	D	1131	1.45	AUG	M	889	1	ACC	T	628	1.04
GAA	E	1336	1.39	AAC	N	798	0.6	ACG	T	412	0.68
GAG	E	588	0.61	AAU	N	1863	1.4	ACU	T	701	1.16
UUC	F	1579	0.83	CCA	P	790	1.28	GUA	V	654	1.18
UUU	F	2235	1.17	CCC	P	605	0.98	GUC	V	450	0.81
GGA	G	816	1.44	CCG	P	386	0.62	GUG	V	377	0.68
GGC	G	347	0.61	CCU	P	694	1.12	GUU	V	741	1.33
GGG	G	543	0.96	CAA	Q	1033	1.4	UGG	W	677	1
GGU	G	562	0.99	CAG	Q	445	0.6	UAC	Y	695	0.65
CAC	H	375	0.58	AGA	R	1136	2.04	UAU	Y	1452	1.35
CAU	H	926	1.42	AGG	R	624	1.12				
AUA	I	1523	1	CGA	R	582	1.05				

*stop codon.

### Comparative chloroplast genomic analysis

**Figure 4:**
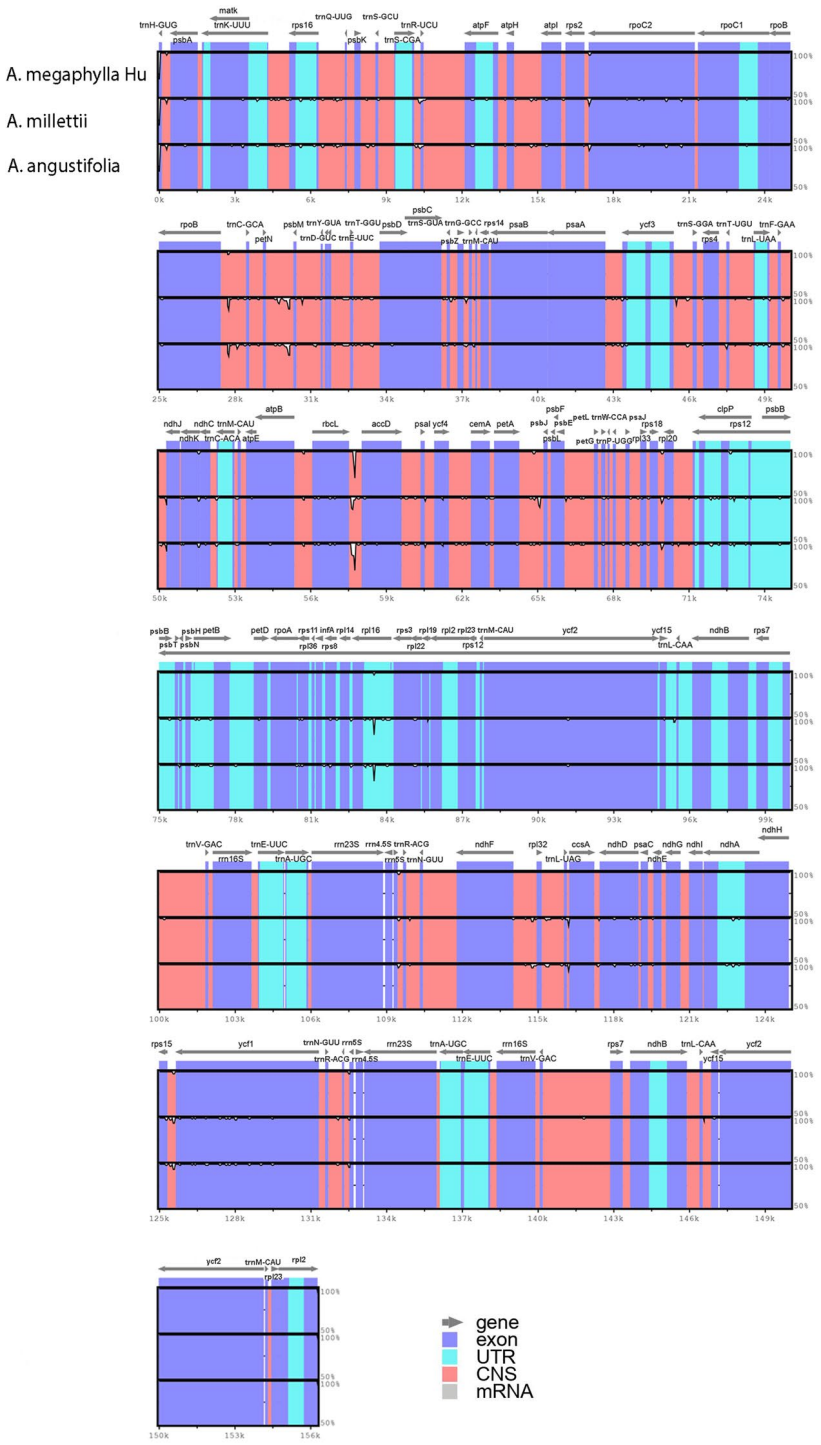
Identity plot comparing the chloroplast genomes of three *Adinandra* species.

To characterize genome divergence, the annotation of *A. megaphylla* Hu was taken as references. The comparison revealed that three chloroplast genomes were highly similar (Fig. 4). The plastome sequences were fairly conserved across the three data with a few regions with a variation. The results exhibited the divergence in LSC and SSC regions were higher than in IR regions. Besides, the sequences in the coding regions tended to be more conserved whereas most of the variations detected were found in conserved non-coding sequences (CNS). The sequences of exons were nearly identical throughout the three taxa. Among the coding genes, the highly disparate regions included *matK, rpoC2, ndhK, ndhD, ycf1.*

**Figure 5:**
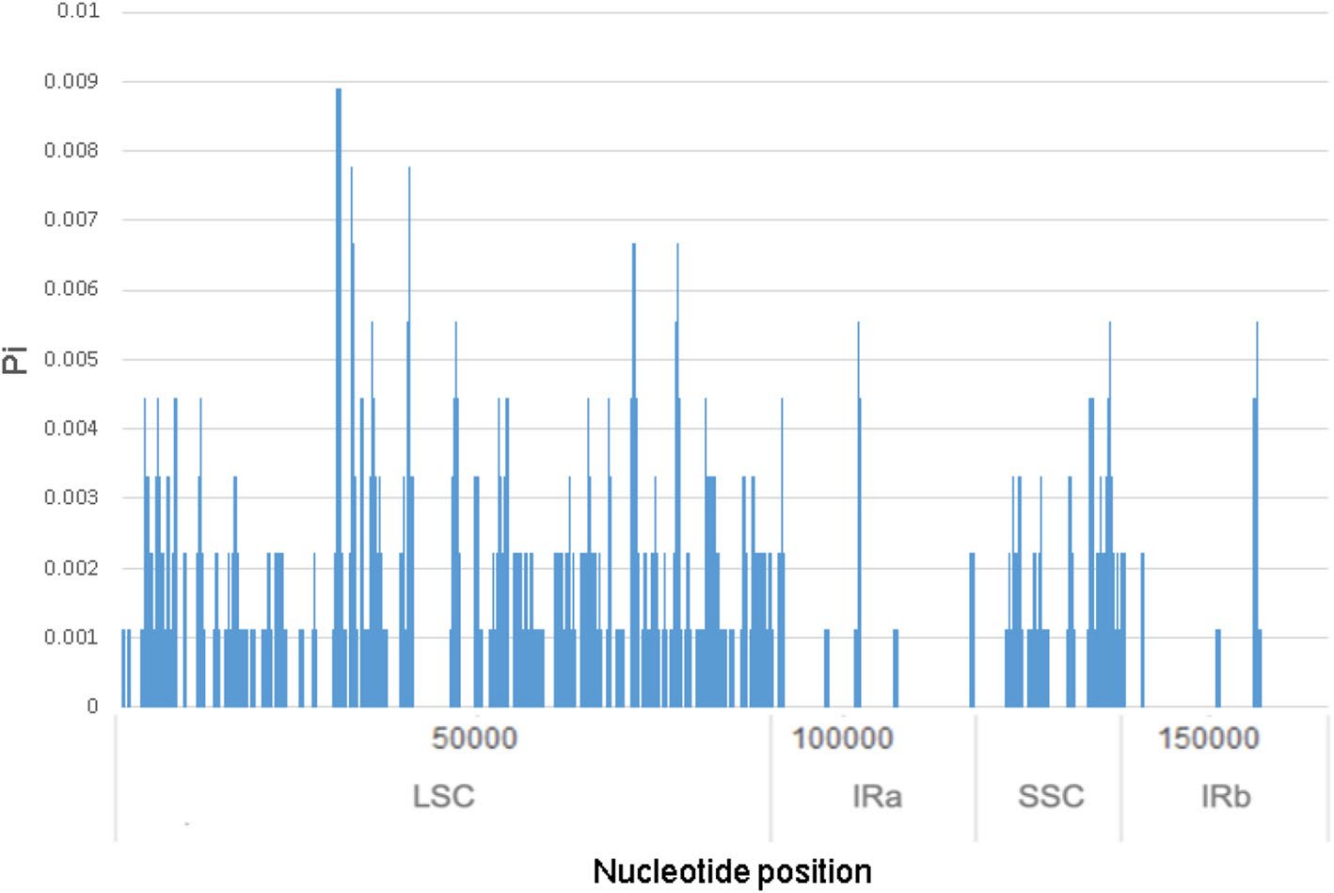
Comparative analysis of nucleotide diversity (Pi) values among the three *Adinandra* species cp genome sequences.

The sliding window analysis showed that the average pi value of the LSC (Pi = 0.001569) and SSC (Pi = 0.001339) regions was much higher than that in the IR (Pi = 0.000219) regions, which showed that LSC and SSC regions contained the most of the variation (Fig. 5). Among the 3 *Adinandra* species, the average value of nucleotide diversity (Pi) was 0.00119.

### IR contraction and expansion in the chloroplast genome

**Figure 6:**
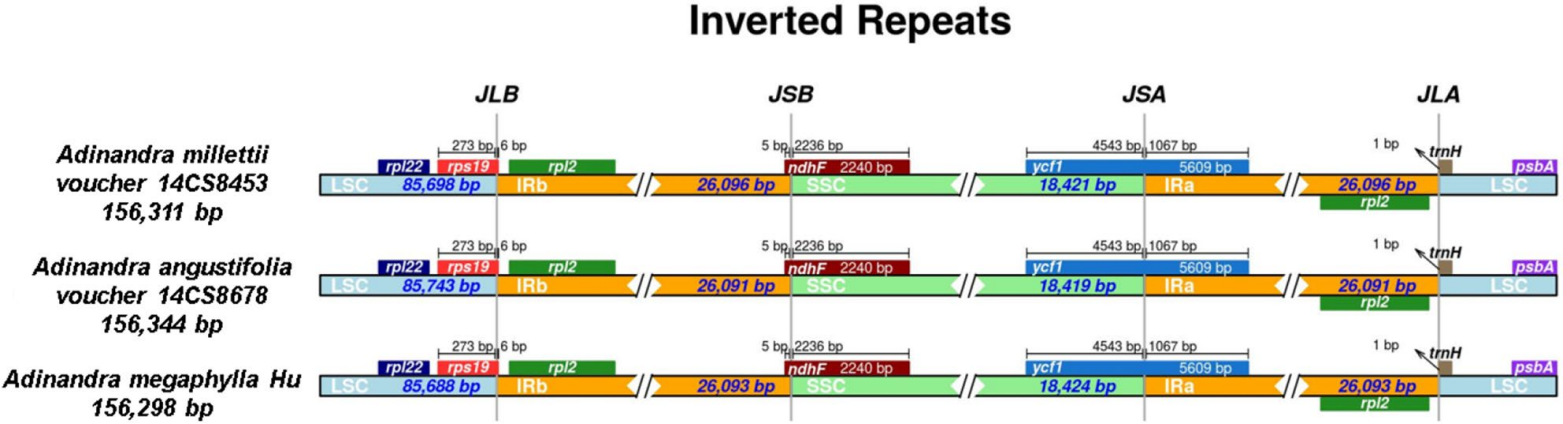
Comparison of LSC, IR and SSC junction positions among the three chloroplast genomes. JLB (junction IRb/LSC), JSB (junction IRb/SSC), JSA (junction IRa/SSC), JLA (junction IRa/LSC).

The IR and SC boundaries of the three *Adinandra* were compared. Overall, the results indicated that the size*,* organization and gene content of the chloroplast genomes were highly similar among the three species. The size of IR ranges from 26,089 bp (*A. megaphylla* Hu*)* to 26,095 bp (*A. millettii)*. And the size of IR of *A. angustifolia* was 26,092 bp. The *ndhF* gene was situated within the LSC region with a 5 bp overlap with the IRa for all three *Adinandra* species. Similarly, the *rps19* gene was positioned within the LSC region with a 6 bp overlap with the IRb. The border across IRa and SSC was located in the region of the *ycf1* gene with 1067 bp tail section of the gene placed in the IRa (Fig. 6). Results of the IR analysis witnessed neither expansion nor contraction of IR regions in the three species.

### Phylogenetic inference

**Figure 7:**
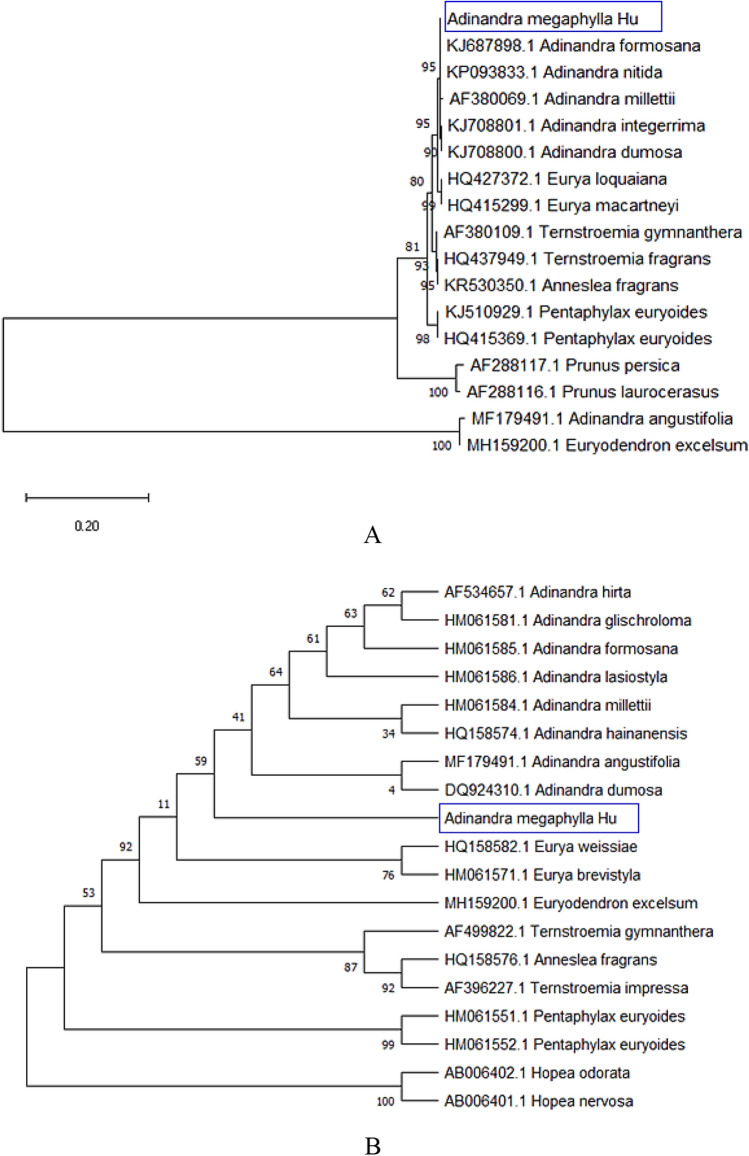
Phylogenetic relationship was inferred using the Maximum Likelihood method based on *matK* (**A**) and *trnL* (**B**) genes.

As shown in Fig. 7A, the phylogenetic analysis was based on *matK* sequences recovered good resolution among genera. In the Pentaphylacaceae, *Euryodendron* and *Adinandra angustifolia* separates outside other genera. The clade of genus *Ternstroemia* and *Anneslea* were sisters to the clade of *Adinandra* and *Eurya* genera. Indeed, all six *Adinandra* species are grouped in one clade, which is divided into three subclades with 95% support; *A. millettii* stood alone in one subclade, *A. integerrima* and *A. dumosa* formed the second subclade, three other species separated into the third one. These results were different from the previous study^15^, in which phylogenetic analysis of *A. angustifolia*, *A. millettii*, *Anneslea fragan* and *Ternstroemia gymnanthera* inferred from the LSC dataset indicated that they belong to one clade (bootstrap values = 100%). This difference might be due to the shortcoming of indicates in phylogenetic analysis when only these four species were representatives of the Pentaphylacaceae appearing in Zhang et al.’s study. In contrast with the *matK* sequence, the *trnL* region dataset yielded less phylogenetic resolution than the bootstrap value was 59% at the clade of the genus *Adinandra* (Fig. 7B)*.* Additionally, the *Adinandra* was separated into six subclades; one constructed by the studied *A. megaphylla* Hu, *A. formosana* and *A. lasiostyla* constructed two distinct subclades. *A. hirta* and *A. glischroloma*; *A. millettii* and *A. hainanensis*; *A. angustifolia* and *A. dumosa* formed three separated subclades, respectively (Fig. 7B). In the case of barcoding among the Pentaphylacaceae family, the *matK* sequence is suggested for better phylogenetic resolution.

## Discussion

Pentaphylacaceae is a family of flowering plants and contains 12 genera including approximately 345 species over the world^16^. A total of 8 cp genomes in the Pentaphylacaceae family have been published currently, 2 of which belong to *Adinandra*. The genus *Adinandra* consists of about 85 species mainly distributed in Bangladesh, Cambodia, China, India, Indonesia, Southern Japan, Laos, Malaysia, Myanmar, New Guinea, Philippines, Sri Lanka, Thailand, and the African tropical forest^17^. Because of bioactive compounds, many species in the genus *Adinandra* are of interest^18–23^.

In the present study, we recently sequenced whole cp genomes for one Vietnamese.

*Adinandra megaphylla* Hu and implemented comparative analyses on three *Adinandra* cp genomes to explore the structure of cp genomes in the taxa. Gene organization together with codon usage patterns was characterized and results indicated the high conservation, which can be helpful for phylogenetic and population genetics studies.

Angiosperm chloroplast genomes have a highly conserved structure and gene content^24,25^. Roughly 129 genes are usually found across the angiosperm chloroplast genomes, among which 18 genes include introns. The analyzed *Adinandra* chloroplast genomes specified the typical quadripartite structure and showed the expected size range (~ 15.6 kb) for angiosperm plants and the conserved gene contents^25,26^. Our gene annotation results were similar to the genetic properties of angiosperm chloroplast genomes. The number of genes present in the cp genome from *A. megaphylla* Hu was 131 and there 18 genes related to introns.

Apart from the two copies of inverted repeats, 48 small repeats were spread out within coding and non-coding regions of the three *Adinandra* taxa. The repeat numbers are not remarkably higher but comparable to other counterparts (the number of dispersed repeats: 49 in *Papaver* spp.; 21 in *Paris* spp.; 36 in *Passiflora*; 37 in *Aconitum*)^27–29^. Repeats are highly associated with the plastome reconstruction in several angiosperm taxa and can be considered as an indication of recombination^30^. Due to the potential to generate secondary structures, repeated sequences can act as recognition signals during the recombination process^31^. It is supposed that recombination rarely occurs in angiosperms because of the predominance of uniparental inheritance. Nevertheless, evidence of intermolecular homologous recombination in flowering plants has been reported^32,33^. To date, studies screening plastome recombination in the taxa are entirely lacking. There was no research demonstrating the presence of plastome recombination in Pentaphylacaceae. In this study, the higher number of repeats in comparison with previous estimates might not be substantiation for inter- and intra-specific plastome recombination.

In terms of constructing phylogenetic relationships of plants, complete chloroplast genomes contribute adequate information and have proven their effectiveness in the capability of classification in lower taxonomic levels^34,35^. *matK* is one of the common DNA barcodes used in plants^36^. However, the phylogeny results indicated that using only a single gene for species classification may generate different results from different genes. The combination of these barcodes can lead to better species identification.

## Conclusion

In this study, three complete chloroplast genomes of *Adinandra* were investigated, including one firstly sequenced chloroplast genomes (*A. megaphylla* Hu*)* comparatively analyzed with other published genus in the family of Pentaphylacaceae for the first time. We assemble the complete chloroplast genome of *A. megaphylla* Hu with 156,298 bp. The structure and gene content of the chloroplast genome of three *Adinandra* were similar and appeared highly conserved. Finally, the phylogenetic relationships built for species of Pentaphylacaceae, in terms of comparison public date with our novel sequence of *Adinandra* species. This study provides the potential of chloroplast genome sequences for enhancing species classification and phylogenetic research for in-depth study within Pentaphylacaceae.

## Material and methods

### Sample collection

**Figure 8:**
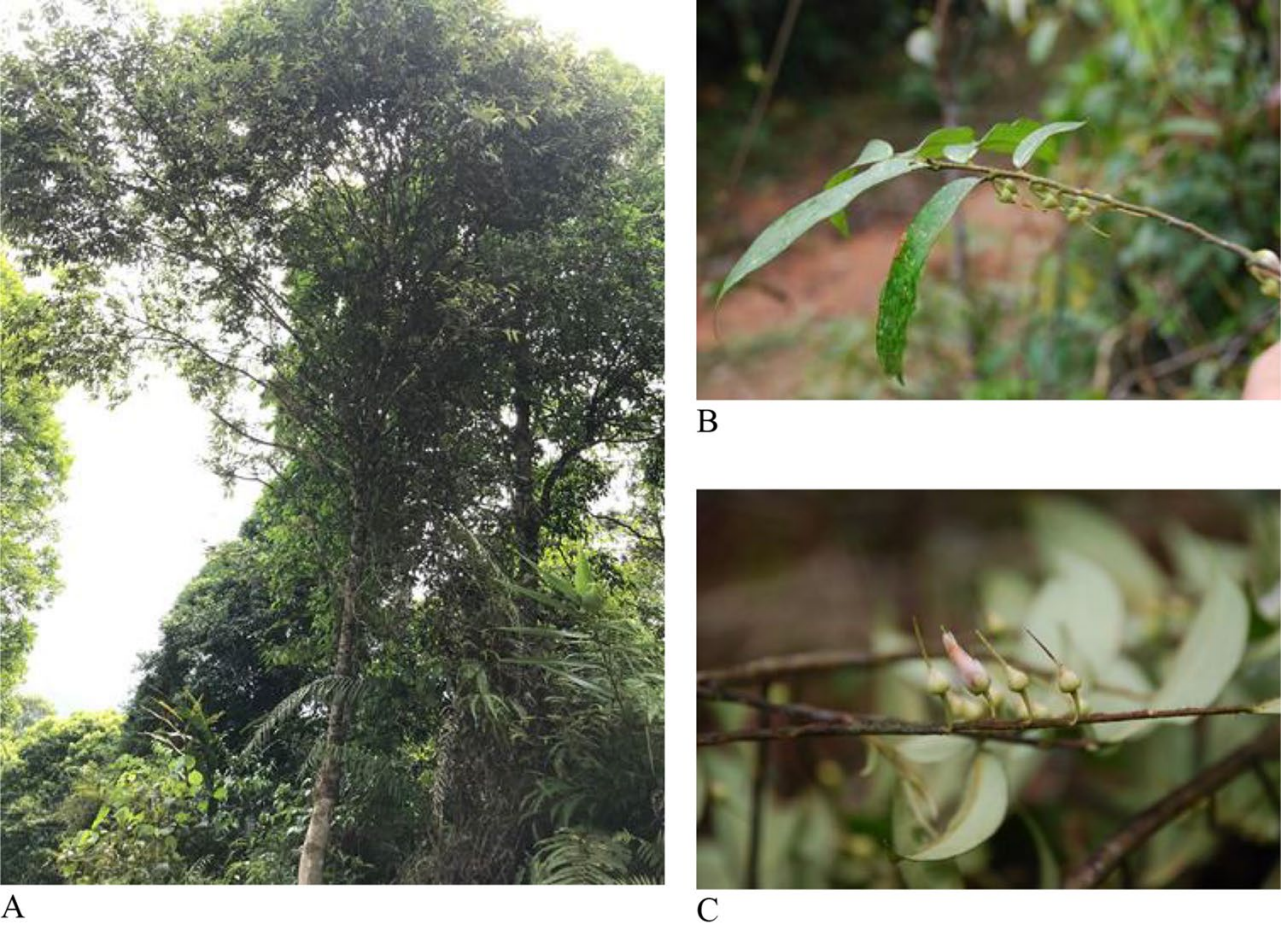
Morphological characteristic of *A. megaphylla* Hu. (**A**) Habit; (**B**) flowering twig; (**C**) bud and flower; Photos by Huu Quan Nguyen.

Samples were collected in Hoang Lien—Van Ban Nature Reserve that belongs to Liem Phu Commune, Van Ban District, Lao Cai Province, Vietnam in August 2019 (code number: Nguyen Huu Quan 01), 1200 m, 21°59′15″N; 104°19′28″E. The taxonomic identification is authenticated by Associate Professor Danh Thuong SY, the head of Botany Department in Faculty of Biology, Thai Nguyen University of Education; the voucher specimens were placed in the Herbarium of the Institute of Ecology and Biological Resources (HN), Hanoi, Vietnam. Fresh leaves with the same code number were used to extract genomic DNA (Fig. 8).

The collection of plant materials has complied with relevant institutional of Hoang Lien—Van Ban Nature Reserve, Vietnamese and international guidelines and legislation.

### DNA extraction and chloroplast genome sequencing

Genomic DNA was extracted from young plant leaves using a modified CTAB method^37^. A260/280 and A260/A230 ratios were measured with the Shimadzu Biospec Nano to assess DNA sample purity. The accurate concentration of double-stranded DNA was determined with Qubit 3 Fluorometer and Qubit HS DNA reagents. Genomic DNA integrity was assessed by agarose gel electrophoresis with 0.8% agarose. Also, DNA libraries were prepared from total genomic DNA using SMRTbell Express Template Prep Kit 2.0 (Pacific Biosciences, Menlo Park, CA), and adapter ligation was subsequently performed, following the manufacturer’s protocol for genomic DNA above 20 kb (Pacific Biosciences). SMRTbell libraries were loaded on one chip and sequenced on a Pacbio SEQUEL system at the Key Laboratory for Gene Technology, Institution of Biotechnology (Hanoi, Vietnam).

### Genome assembly and annotation

The total gDNA was sequenced in the PacBio platform by the resequencing method. The sequences derived from the cp genome were identified via the local Blast program^38^ using *Adinandra angustifolia* (MF179491) cp genomes as the reference^15^. Subsequently, the software HGAP4^39^ was used to assemble the cp genome. The protein-coding, rRNA, and tRNA genes were annotated by the CpGAVAS pipeline^40^. The tRNAscan-SE ver. 1.21 software^41^ was applied to verify the tRNA genes with default parameters. The OrganellarGenomeDRAW tool (OGDRAW) ver. 1.3.1^42^ was selected to create the circular gene map. Repeat elements were found using two approaches. Web-based simple sequence repeats finder MISA-web^43^ was used to detect microsatellites, including 10 repeat units for mono-, 5 repeat units for di-, 4 repeat units for tri-, and 3 repeat units for tetra-, penta-, and hexa-nucleotide SSRs. Among the SSRs of each type, comparing the size of SSRs was employed to count the polymorphic SSRs among the three species. The size and type of repeats in the three *Adinandra* plastomes were investigated using REPuter^44^ with the set parameters as follows: a minimal repeat size of 20 bp, hamming distance of 3 kb, and 90% or greater sequence identity.

### Genome comparison

For comparative purposes, we collected two available cp genomes of *A. angustifolia* (#MF179491) and *A. millettii* (#MF179492) from GenBank (https://www.ncbi.nlm.nih.gov/genbank/). The overall genome structure, genome size, gene content and repeats across all three *Adinandra* species were compared^15^. The whole plastome sequences of the three *Adinandra* plants were aligned with the MAFFT server^45^ and visualized using LAGAN mode in mVISTA^46^. For the mVISTA plot, we used the annotated cp genome of *A. megaphylla* Hu as a reference. The Irscope^47^ was employed to visually display and compare the borders of large single-copy (LSC), small single-copy (SSC), and inverted repeat (IR) regions among the three *Adinandra* species. We also determined the codon usage bias and the sequence divergence among the three *Adinandra* species through a sliding window analysis computing pi among the chloroplast genomes in DnaSP ver. 6.12.03^48^. For the sequence divergence analysis, we applied the window size of 600 bp with a 200 bp step size.

### Phylogenetic identification

The sequences of *matK* and *trnL* from all *Adinandra* species and other members of the family Pentaphylacaceae from Genbank (https://www.ncbi.nlm.nih.gov/genbank/) were used to identify the taxonomic position of the studied *A. megaphylla* Hu. These sequences were aligned with ClustalW mode in Unipro UGENE software v36.0^49^ before a maximum likelihood (ML)^50^ phylogenetic tree was constructed using Mega-X software^51^ with 1000 bootstraps. The chosen methods followed the previous study of this genus^15^.
